# Variation in the utilization of angioembolization for splenic injury in hospitals: a nationwide cross‐sectional study in Japan

**DOI:** 10.1002/ams2.837

**Published:** 2023-04-12

**Authors:** Makoto Aoki, Toshikazu Abe, Shuichi Hagiwara, Daizoh Saitoh

**Affiliations:** ^1^ Advanced Medical Emergency Department and Critical Care Center Japan Red Cross Maebashi Hospital Maebashi Japan; ^2^ Department of Emergency and Critical Care Medicine Tsukuba Memorial Hospital Tsukuba Japan; ^3^ Department of Health Services Research University of Tsukuba Tsukuba Japan; ^4^ Department of Emergency Medicine Kiryu Kosei General Hospital Kiryu Japan; ^5^ Division of Traumatology Research Institute, National Defense Medical College Tokorozawa Japan

**Keywords:** Angioembolization, angiography, splenic injury, trend, variation

## Abstract

**Aim:**

Substantial variations in the utilization of angioembolization have been reported internationally. However, the existence of variations in the utilization of angioembolization in Japan is currently unknown.

**Methods:**

This was a cross‐sectional study using data from a nationwide trauma registry in Japan. Of the 4,896 registered adult patients with splenic injury, we investigated 3,319 patients in the top 25% of the hospitals that registered the highest number of splenic injury patients in the Japan Trauma Data Bank. The primary outcome of this study was initial angioembolization. We calculated the expected initial angioembolization rates using multiple regression analysis adjusted for patient factors. In addition, we evaluated the range of observed‐to‐expected initial splenic angioembolization ratio for each hospital. Moreover, we assessed whether this ratio was increased with time.

**Results:**

The frequency of initial splenic angioembolization ranged from 0% to 52%. The median expected initial angioembolization rate, calculated through multiple logistic regression analysis, was 19.7%. The observed‐to‐expected initial splenic angioembolization ratio for each hospital ranged from 0 to 2.36. The observed initial angioembolization rate tended to increase with time (*P* < 0.001).

**Conclusions:**

Despite adjustment for patient factors, substantial variations were observed in the utilization of splenic angioembolization among hospitals in Japan.

## Graphical abstract

This nationwide cross‐sectional study examined the use of angioembolization for splenic injury in Japan. Despite adjustment for patient factors, substantial variations were observed in the utilization of splenic angioembolization among hospitals in Japan. Higher accessibility to computed tomography and shorter time to angiography were related to increased utilization of splenic angioembolization.

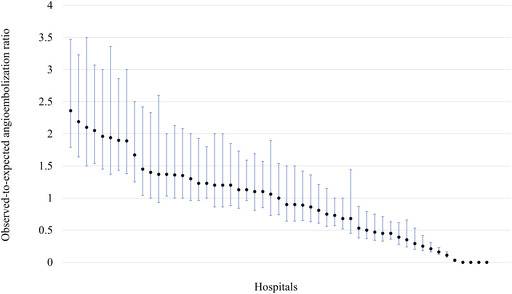

## INTRODUCTION

Blunt abdominal organ injury occurs in approximately 30% of patients with polytrauma,^1^ and is very common in patients with trauma in Japan.^2^, ^3^ Particularly, splenic injury is one of the most frequent injuries in abdominal organs.^1^, ^2^, ^3^ The management of blunt splenic injury has drastically changed since the introduction of nonoperative management (NOM).^4^ Previous studies reported that approximately 80%–90% of patients with splenic injury could be treated by NOM.^5^, ^6^ Angioembolization is used with NOM for the treatment of adult blunt splenic injuries in adults, and its utilization has been increasing.^6^ Moreover, recent guidelines recommended its use in hemodynamically stable patients with contrast blush.^7^

Substantial variations in the utilization of angioembolization have been reported internationally.^8^, ^9^, ^10^ In addition, accessibility to interventional radiology remains limited even in level 1 trauma centers in the United States. In Japan, the existence of variations in the utilization of angioembolization is currently unknown. The utilization of splenic angioembolization could be affected by patient characteristics and hospital factors. The latter factors include institutional protocols, trauma practitioner skills, and/or hospital diagnostic methods, such as computed tomography (CT) and angiography suite. Therefore, this study aimed to investigate variation in the utilization of splenic angioembolization in Japan.

## MATERIALS AND METHODS

### Study design

In this cross‐sectional study, data from the Japan Trauma Data Bank (JTDB) were analyzed to determine the existence of variation in the utilization of angioembolization for splenic injury in hospitals. This study was approved by the medical ethics committee of the Japan Red Cross Maebashi Hospital.

### Patient selection

Data were obtained from the JTDB, a nationwide trauma registry established in 2003 by the Japanese Association for the Surgery of Trauma and the Japanese Association for Acute Medicine to ensure and improve the quality of trauma care in Japan. During the study period (2004–2018), a total of 291 hospitals, including 95% of tertiary emergency medical centers in Japan, contributed to the JTDB.^11^ In this study, adult patients (aged ≥ 16 years) with blunt splenic injury were selected according to the Abbreviated Injury Scale (AIS) codes of splenic injury (Table S1). Subsequently, the number of patients with splenic injury at each hospital was confirmed, and the top 25% of the hospitals that registered the highest number of splenic injury patients in the JTDB were included in the analysis.

### Data collection

The JTDB collects 92 data elements related to patient and hospital information, such as demographics, transfer process, vital signs at hospital arrival (e.g., systolic blood pressure, heart rate, respiratory rate, and Glasgow Coma Scale), trauma scores (e.g., injury severity score), diagnostic procedures, initial treatment procedures, and in‐hospital survival. Regarding initial treatment procedures, the use of angioembolization (including abdominal angioembolization) and laparotomy (including splenectomy) was recorded in the JTDB. Initial splenic angioembolization was defined by the code of initial angioembolization and abdominal organ embolization among patients with splenic injury. Hospital codes were registered as anonymous numbers in the JTDB.

### Study outcomes

The primary outcome of this study was the initial utilization of splenic angioembolization. The secondary outcomes were transfusion rates, total length of hospital stay, and in‐hospital mortality.

### Statistical analysis

Continuous variables were expressed as medians (interquartile range), while categorical variables were presented as counts and percentages. First, we produced a histogram of utilization of initial splenic angioembolization at each hospital. Second, we calculated the expected initial angioembolization rates among the included patients using multiple regression analysis. Variables for the estimation of initial angioembolization were selected based on scientific rationale and statistically significant associations. The included variables were age, sex, vital signs at hospital arrival (e.g., systolic blood pressure, heart rate, respiratory rate, and Glasgow Coma Scale), AIS of splenic injury, and Injury Severity Score. Third, we calculated the observed‐to‐expected initial splenic angioembolization ratio for each hospital and depicted it using caterpillar plots. Fourth, we evaluated the trends of observed and expected initial angioembolization rates. The trends were evaluated using the Cochran–Armitage trend test. Fifth, we calculated the median expected initial splenic angioembolization rates, and categorized the institutions as low‐ and high‐embolization hospitals according to the cutoff value stated in relevant literature.^9^ Finally, we compared the patient characteristics and outcomes between low‐ and high‐embolization hospitals. Furthermore, we assessed the characteristics of patients who underwent initial splenic angioembolization.

A two‐sided *P*‐value <0.05 with a 95% confidence interval denoted statistically significant differences. Statistical analyses were performed using the R software (version 3.5.2; R Foundation for Statistical Computing, Vienna, Austria).

## RESULTS

**Fig. 1:**
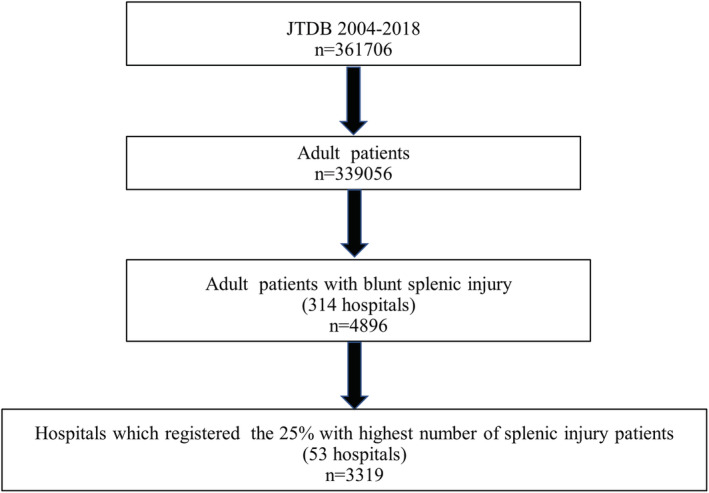
Flowchart of patients included in this study. JTDB, Japan Trauma Data Bank.

A total of 361,706 patients were registered in the JTDB from 2004 to 2018 (Fig. 1). Among 339,056 adults, 4,896 patients had blunt splenic injury. The histogram (Fig. S1) illustrates the number of adult patients with blunt splenic injury registered to the JTDB by the top 25% of the hospitals. Of those, 3,319 adult patients with blunt splenic injury from 53 hospitals (out of 314 hospitals) were included in the analysis.

The frequencies of initial splenic angioembolization ranged from 0% to 52% (Fig. S2). The median expected initial angioembolization rate, calculated by multiple logistic regression analysis, was 19.7%.

**Fig. 2:**
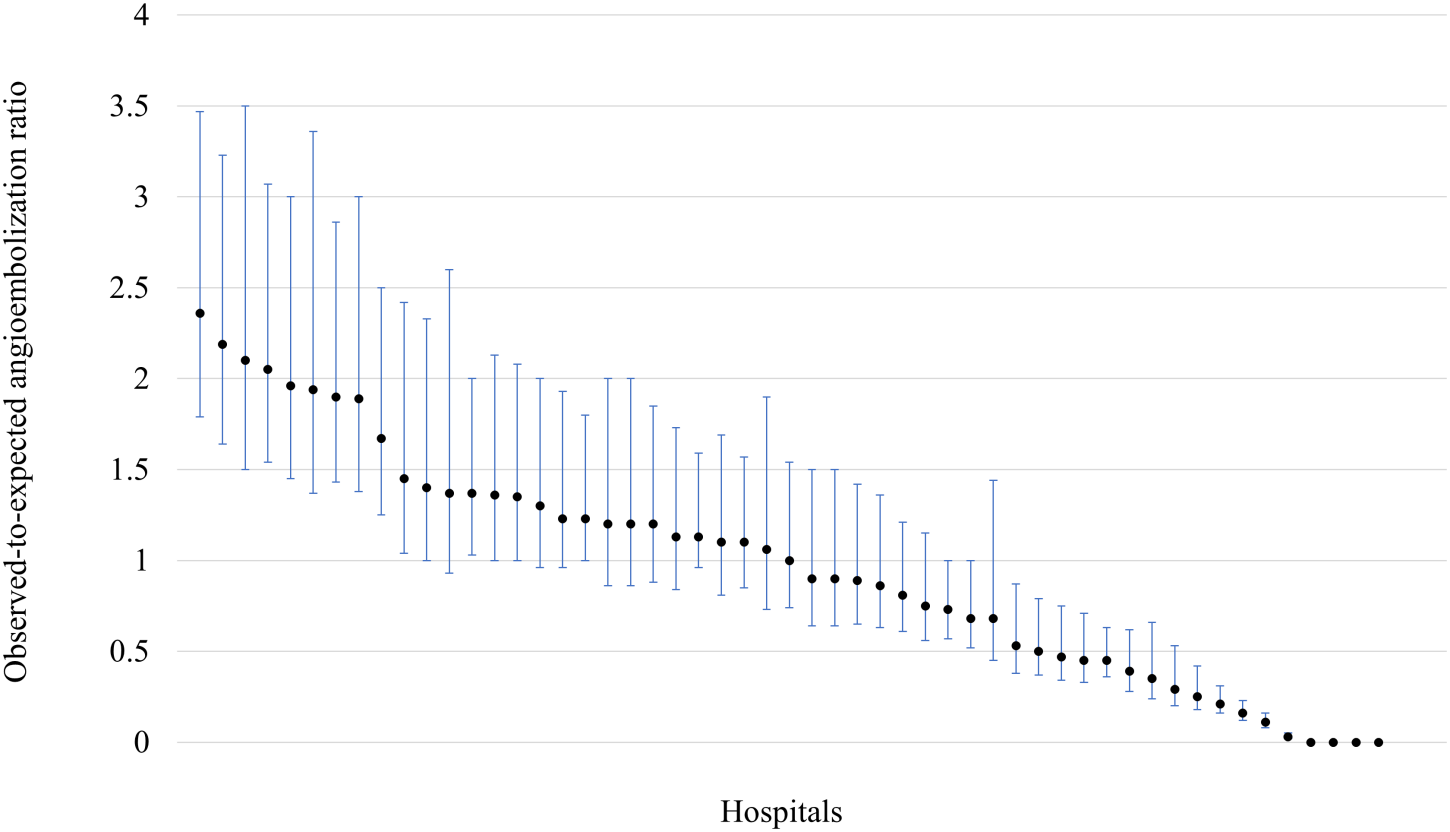
Caterpillar plots for the observed‐to‐expected angioembolization ratio among patients with splenic injury in included hospitals.

**Fig. 3:**
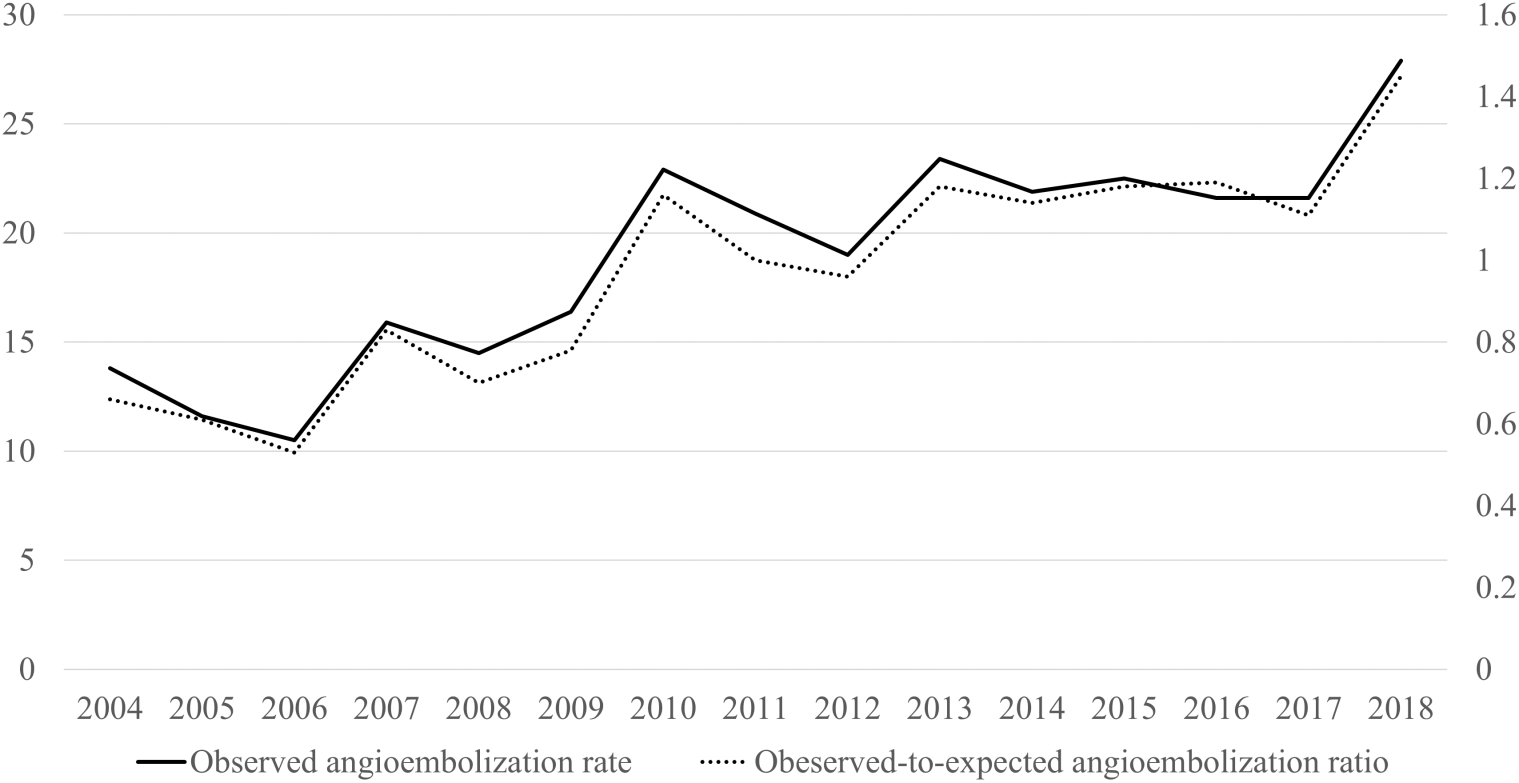
Trends of observed angioembolization rate and observed‐to‐expected angioembolization ratio.

The observed‐to‐expected initial splenic angioembolization ratio for each hospital ranged from 0 to 2.36. The distribution of the observed‐to‐expected initial splenic angioembolization ratio for each hospital is shown in Figure 2. The observed initial angioembolization rate tended to increase with time (*P* < 0.001; Fig. 3).

The cutoff rate determining low‐ and high‐angioembolization hospitals was set at 20%. The median number of patients at each hospital between the low‐ and high‐angioembolization groups was 59 patients (26 hospitals) and 66 patients (27 hospitals), respectively. The characteristics of low‐ and high‐angioembolization hospitals are shown in Table 1; there were no remarkable differences noted in these characteristics. Table 2 shows management and outcomes in low‐ and high‐angioembolization hospitals. Initial CT assessment was more frequent and the time to CT was shorter in high‐angioembolization hospitals compared with low‐angioembolization hospitals. Regarding outcomes, lower in‐hospital mortality was recorded in high‐angioembolization hospitals versus low‐angioembolization hospitals. Table 3 presents the characteristics and outcomes among patients who underwent initial angioembolization in low‐ and high‐angioembolization hospitals. There were no remarkable differences noted in these parameters between the two categories of hospitals. Observed‐to‐expected initial splenic angioembolization ratio between low‐ and high‐angioembolization hospitals tended to increase with time (*P* = 0.001 and *P* < 0.001).

**Table 1:** Population characteristics

Variable	Low embolization, *n* = 1,533	High embolization, *n* = 1,786	*P*‐value
Demographics				
Age (years), median (IQR)	43 (24–64)	41 (23–63)	0.061
Sex, male, *n* (%)	1,123 (73)	1,306 (73)	0.937
Transfer process, *n* (%)				
Direct	1,199 (79.2)	1,377 (77.9)	0.349
Indirect	314 (20.8)	391 (22.1)	
ISS, median (IQR)	26 (17–36)	25 (16–36)	0.254
Head AIS score > 2, *n* (%)	365 (24)	452 (24)	0.332
Chest AIS score > 2, *n* (%)	995 (65)	1,115 (63)	0.138
Abdomen AIS score > 2, *n* (%)	1,025 (67)	1,248 (70)	0.072
Spleen injury grade, median (IQR)	3 (2–3)	3 (2–3)	0.163

AIS, Abbreviated Injury Scale; IQR, interquartile range; ISS, Injury Severity Score.

**Table 2:** Management and outcomes of blunt spleen injury

Outcomes	Low embolization, *n* = 1,533	High embolization, *n* = 1,786	*P*‐value
Initial CT examination, *n* (%)	1,262 (82.3)	1,598 (89.5)	<0.001
Time to CT, *n* (%)	39 (26–64)	29 (20–44)	<0.001
Injury management, *n* (%)			
Initial SAE	116 (7.6)	558 (31)	<0.001
Initial splenectomy	234 (15)	219 (12)	0.013
Splenectomy after SAE	6 (0.4)	22 (1.2)	0.012
Delayed splenectomy	17 (1.1)	32 (1.8)	0.114
Outcomes			
Transfusion, *n* (%)	767 (52)	907 (52)	0.860
Total length of stay (days), median (IQR)	15 (7–33)	17 (8–34)	0.062
In‐hospital mortality, *n* (%)	273 (18)	254 (15)	0.005

CT, computed tomography; IQR, interquartile range; SAE, splenic artery embolization.

**Table 3:** Subgroups of patients who underwent initial angioembolization

	Low embolization, *n* = 116	High embolization, *n* = 558	*P*‐value
Arrival time, *n* (%)			
Daytime	68 (59.6)	336 (60.8)	0.834
Nighttime	46 (40.4)	217 (39.2)	
Physiology, *n* (%)			
Shock	36 (31.0)	160 (28.8)	0.654
GCS < 8	11 (9.6)	51 (9.4)	
Trauma severity, *n* (%)			
Spleen AIS ≥ 3	87 (75.0)	223 (75.8)	0.145
ISS	23 (17–34)	24 (14–34)	0.655
Time to angioembolization (min), median (IQR)	123 (90–206)	95 (65–147)	<0.001
Outcome			
Transfusion, *n* (%)	55 (57.9)	302 (54.9)	0.605
Total length of stay (days), median (IQR)	20 (12–35)	17 (11–31)	0.158
In‐hospital mortality, *n* (%)	10 (8.8)	40 (7.4)	0.565

AIS, Abbreviated Injury Scale; GCS, Glasgow Coma Scale; IQR, interquartile range; ISS, Injury Severity Score.

## DISCUSSION

### Brief summary

This nationwide, cross‐sectional study analyzed data from the JTDB. The results demonstrated substantial variation in the use of initial angioembolization for the treatment of splenic injury in adult patients in hospitals. The rate of utilization of angioembolization for each hospital ranged from 0% to 52%. The observed‐to‐expected angioembolization ratio increased with time and almost doubled from 2004 to 2018.

### Interpretation of the present findings

The median rate of initial splenic angioembolization was approximately 20%. This rate is comparable to that recently reported by level 1 trauma centers in the United States (17%). Substantial variation in the utilization of initial splenic angioembolization has been reported in the United States.^8^, ^9^, ^10^ The rate of initial splenic angioembolization substantially differed among institutions in Japan, despite adjustment for patient factors. The selection of initial splenic angioembolization may be affected by various factors in each facility, such as institutional protocols, availability of interventional radiology, and equipment. A survey from the British Society of Interventional Radiologists revealed substantial differences in institutional protocols which followed guidelines, as well as the physiology and splenic injury grades of patients.^12^ A previous report from level 1 trauma centers in the United States identified that the use of angioembolization differed according to the splenic AIS grade between low‐ and high‐embolization hospitals.^8^ The present study did not detect statistically significant differences in terms of patient physiology and trauma severity between low‐ and high‐embolization hospitals. Thus, patient factors may not affect the selection of initial angioembolization.

From the perspective of availability of interventional radiology, the R Adams Cowley Shock Trauma Center reported that a 24/7 on‐call service for emergent endovascular intervention could increase its rate of utilization.^13^ However, the selected top 25% of hospitals in this study did not exhibit differences in terms of on‐call service for interventional radiology.^14^ Therefore, there was no significant difference observed in the utilization of angioembolization according to the time of patient arrival.

The substantial variation observed in the utilization of initial splenic angioembolization may be due to accessibility to CT and/or angiography suite. We detected significant differences in the rates of initial CT examination, door‐to‐CT time, and door‐to‐angioembolization time. In the current guidelines,^7^, ^15^ the presence of contrast blush on enhanced CT is an indication for interventional radiology, and earlier accessibility to CT could contribute to increased utilization of angioembolization.^16^ In addition, the technological advancement of hybrid theater enabled clinicians to simultaneously perform CT scanning and angiography, and increased the rate of angioembolization.^17^

Another finding of this study was that the utilization of splenic angioembolization was increased with time, and the rate was raised from 12.0% to 22.0% between 2004–2006 and 2014–2016. Similar trends were reported from level 1 trauma centers in the United States^6^ and Taiwan.^18^ Similar to Taiwan,^18^ the top 25% of institutions participating in the JTDB were tertiary, well‐equipped, emergency hospitals. The observed‐to‐expected angioembolization ratio was increased from 0.19 to 0.53 in the low‐embolization group and from 1.13 to 1.62 in the high‐embolization group, respectively, between 2004–2006 and 2014–2016. Although substantial differences remain, the low‐embolization centers gradually improve their capacity for interventional radiology.

Regarding other outcomes, high‐embolization hospitals were associated with significantly decreased in‐hospital mortality rates versus low‐embolization hospitals. The rate of in‐hospital mortality in the subgroup of patients who underwent initial angioembolization did not show a statistically significant difference in mortality between high‐embolization hospitals and low‐embolization hospitals. However, high‐embolization hospitals also tended to have decreased mortality versus low‐embolization hospitals (7.4% versus 8.8%, respectively). Thus far, there are no previous reports demonstrating significant differences in mortality between low‐ and high‐embolization institutions.^8^, ^9^, ^10^

We acknowledge the following limitations of this study. First, the selection of angioembolization and laparotomy may depend on hospital characteristics such as expertise of physicians (e.g., interventional radiology or acute care surgery) and/or availability of CT. Information on hospital factors could not be gained from the JTDB, and we could only adjust patients’ factors. Second, we were unable to identify the exact indication for angioembolization (e.g., contrast blush on CT or other). Besides, we could not distinguish targeted embolized regions for multiple abdominal organ injury due to limited information of the JTDB. Third, we included the top 25% of hospitals that registered adult splenic injury patients in the JTDB. Registration of patients were entrusted to each participated hospital and the top 25% of hospitals were assumed to rigorously register admitted patients. The cutoff percentage of 25% was determined based on distributions of registered patients at each hospital (Fig. S1). Fourth, this study evaluated tendencies in the utilization of initial angioembolization for splenic trauma according to the hospital level, which involves a risk of ecological fallacy. However, there was no ecological fallacy in this study because our hospital data were extracted at the patient level.

Despite adjustment for patient factors, there were substantial variations noted in the utilization of splenic angioembolization among hospitals in Japan. Higher accessibility to CT and shorter time to angiography were related to increased utilization of splenic angioembolization. The utilization of angioembolization in Japan increased with time.

## DISCLOSURE

Approval of the Research Protocol with Approval No. and Committee Name: This study was approved by the medical ethics committee of the Japan Red Cross Maebashi Hospital. Because of the anonymous and retrospective nature of the study, the need for informed consent was waived.

Informed Consent: Not applicable.

Registry and the Registration No. of the Study/Trial: Not applicable.

Animal Studies: Not applicable.

Conflict of Interest: All authors declare that they have no competing interests.

## AUTHORS’ CONTRIBUTIONS

MA and TA conceived and designed this study, interpreted the data, drafted the manuscript, and reviewed the manuscript for important intellectual content. SH and DS interpreted the data and reviewed the manuscript for important intellectual content.

## Supporting information


**Figure S1** Distribution of patients with splenic injury between hospitals. Bar: 75% interquartile range of patients with splenic injury (n = 34).

**Figure S2** Distribution of percentages of angioembolization for splenic injury between included hospitals.

**Table S1** Splenic injury codes of the Abbreviated Injury Scale (1990 revision, updated 1998).
