# Supplementary figures and images for: Variation in the utilization of angioembolization for splenic injury in hospitals: a nationwide cross‐sectional study in Japan
Source: Acute Med Surg. 2023 Apr 12;10(1):e837. doi: 10.1002/ams2.837 (PMC10097635; doi:10.1002/ams2.837)

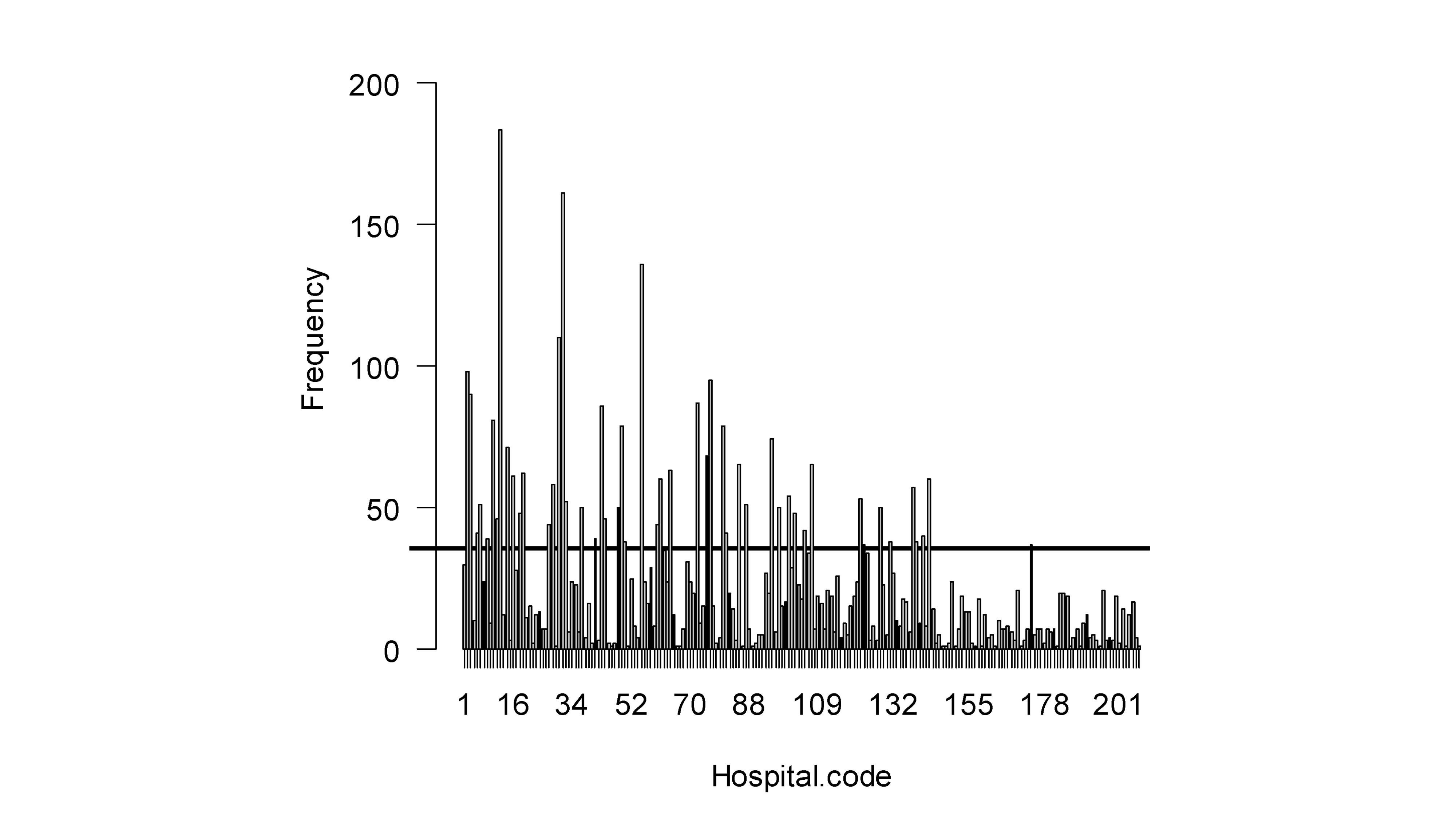

Supplement: Supplementary file 1 — Figure S1 Distribution of patients with splenic injury between hospitals. Bar: 75% interquartile range of patients with splenic injury (n = 34). [file AMS2-10-e837-s002.TIF]

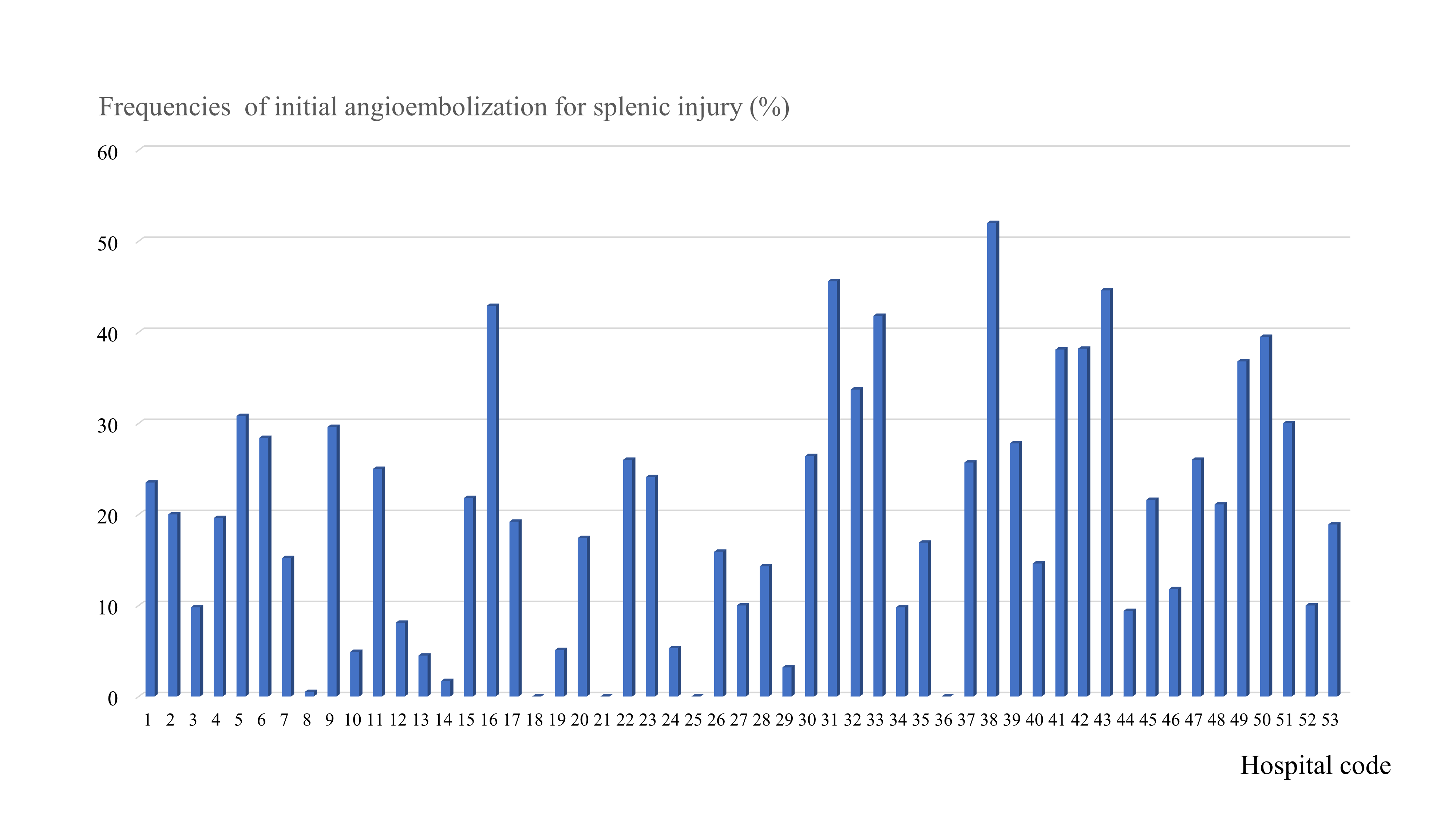

Supplement: Supplementary file 2 — Figure S2 Distribution of percentages of angioembolization for splenic injury between included hospitals. [file AMS2-10-e837-s003.TIF]
