# Supplementary material for: Variation in the utilization of angioembolization for splenic injury in hospitals: a nationwide cross‐sectional study in Japan
Source: Acute Med Surg. 2023 Apr 12;10(1):e837. doi: 10.1002/ams2.837 (PMC10097635; doi:10.1002/ams2.837)
Supplement: Supplementary file 3 — Table S1 Splenic injury codes of the Abbreviated Injury Scale (1990 revision, updated 1998). [file AMS2-10-e837-s001.docx]

Supplemental Table 1.

Splenic injury codes of Abbreviated injury scale 1990 revision updated 98

544299.2

544210.2

544212.2

544214.3

544220.2

544222.2

544224.3

544226.4

544228.5

544240.3
